# Age-related macular degeneration-associated variants at chromosome 10q26 do not significantly alter *ARMS2* and *HTRA1* transcript levels in the human retina

**Published:** 2010-07-15

**Authors:** Atsuhiro Kanda, Dwight Stambolian, Wei Chen, Christine A. Curcio, Gonçalo R. Abecasis, Anand Swaroop

**Affiliations:** 1 Neurobiology Neurodegeneration & Repair Laboratory (N-NRL), National Eye Institute, National Institutes of Health, Bethesda, MD; 2 Department of Ophthalmology and Human Genetics, University of Pennsylvania, Philadelphia, PA; 3 Center for Statistical Genetics, Department of Biostatistics, University of Michigan, Ann Arbor, MI; 4 Department of Ophthalmology, University of Alabama at Birmingham, Birmingham, AL

## Abstract

**Purpose:**

Multiple studies demonstrate a strong association between three variants at chromosome 10q26 – rs10490924, del443ins54, and rs11200638 – near the age-related maculopathy susceptibility 2 (*ARMS2)* and high-temperature requirement factor A1 (*HTRA1)* genes with susceptibility to age-related macular degeneration (AMD). In different reports, the del443ins54 and rs11200638 variants are suggested to affect *ARMS2* mRNA stability and/or *HTRA1* mRNA expression, respectively. The goal of this study is to examine whether these AMD-associated variants alter expression levels of *ARMS2* and *HTRA1* in human retina samples.

**Methods:**

Genomic DNA and total RNA were obtained from 35 human retinas (three young controls, average age=32 years; twenty aged controls, average age=72 years; and twelve AMD retinas, average age=77 years) using standard procedures. As *ARMS2* exhibits higher expression in the human placenta, we also included eighteen placenta samples in our analysis. Four polymorphisms – rs2736911, rs10490924, del443ins54, and rs11200638 – were genotyped by PCR followed by sequencing. Expression of *ARMS2*, *HTRA1* and three endogenous control genes (rRNA [*rRNA*], hypoxanthine phosphoribosyltransferase 1 [*HPRT1*]*,* and glyceraldehyde-3-phosphate dehydrogenase [*GAPDH*]) was measured by real-time quantitative RT–PCR using Taqman gene expression or SYBR Green assays.

**Results:**

*ARMS2* and *HTRA1* mRNA levels did not show a significant difference in expression among the control (young and elderly) and AMD retinas. No association of del443ins54 and rs11200638 variants was detected with mRNA expression levels of *ARMS2* or *HTRA1* in the retina. Human placenta samples showed high variability in expression levels.

**Conclusions:**

We did not find association between AMD susceptibility variants at 10q26 and steady-state expression levels of either *ARMS2* or *HTRA1* in the human retina.

## Introduction

Age-related macular degeneration (AMD) is a prevalent and complex disorder that primarily affects the central region of the retina (macula) and is a leading cause of untreatable visual impairment and legal blindness in older individuals [1,2]. Early stages of AMD exhibit pigmentary and morphological changes in the retinal pigment epithelium (RPE). Large and soft drusen in the macula are identified as strong risk factors for the development of advanced forms of AMD, which include central geographic atrophy and choroidal neovascularization [3-6]. Advanced age, inherited susceptibility involving several genes, and environmental risk factors are involved in the pathogenesis of AMD [6].

Genetic studies have demonstrated the contribution of multiple susceptibility loci; variants on chromosomes 1q31 and 10q26 account for a major part of the genetic susceptibility to AMD [6,7]. Several independent reports have elucidated the importance of Y402H and multiple non-coding variants in the complement factor H (*CFH)* gene on chromosome 1q31 in AMD susceptibility [8-13]. Single nucleotide polymorphisms (SNPs) in three other complement factor genes (*C2/BF*, *C3,* and *CFI*) are also associated with susceptibility to AMD [14-17]. More recent genome wide association studies have also implicated variants near tissue inhibitor of metalloproteinase 3 (*TIMP3)* and several high density lipoprotein (HDL)-associated loci in AMD susceptibility [18,19].

**Figure 1:**
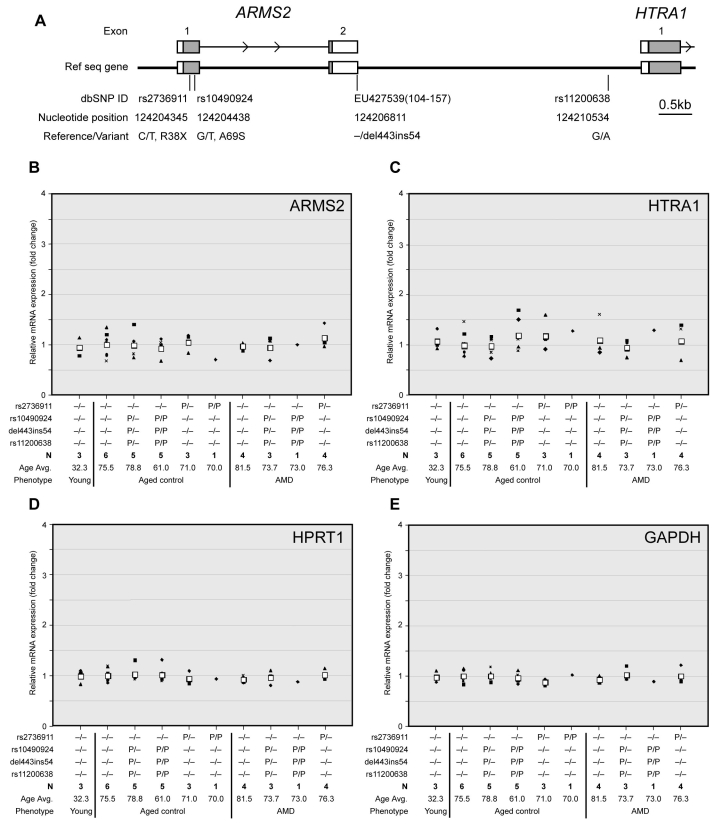
Relationship of *ARMS2* and *HTRA1* expression in the human retina to the genotypes at 10q26. **A**: Schematic representation of positions of polymorphic variants at chromosome 10q26. White boxes indicate exons and gray shows translated regions. Numbers indicate the nucleotide position in NT_030059.12 (NCBI Reference Sequence). **B**-**E**: Gene expression in the human retina. We investigated the effect of AMD-associated variants near *ARMS2* and *HTRA1* genes on expression levels of *ARMS2* and *HTRA1* in human retina samples. Fold changes in expression of the target genes (*ARMS2*, *HTRA1*, *HPRT1*, and *GAPDH*) relative to the internal control gene (*rRNA*) were correlated to the genotypes of four polymorphisms at 10q26. The samples were analyzed after qRT–PCR reactions performed in quadruplicate with Taqman gene expression or SYBR Green assays. The fold change average in expression of the target gene was calculated using the 2^-ddCt^ method; ddCt=(C_t_,Target - C_t_,rRNA)_experiment_ - (C_t_,Target - C_t_,rRNA)_reference_. Gene expressions in wild-type (with common variants at the four loci) older normal retinas were used as a reference. Black unique symbols (e.g., triangle, square, diamond, dash, X’s cross, circle) indicate individual sample changes, and white square shows the average. Abbreviations: Avg. represents average; N represents number of samples with a given genotype; P represents polymorphism (P/P represents homozygous; P/- represents heterozygous change. Variants are in the left column).

Outside the complement pathway, the major genetic contributor to AMD susceptibility is the locus at 10q26. The association signals center over two neighboring genes, *ARMS2* (age-related maculopathy susceptibility 2, previously called LOC387715; OMIM +611313) [20,21] and *HTRA1* (high-temperature requirement factor A1; OMIM 602194) [22,23], suggesting two plausible candidates. The AMD-associated SNP rs11200638 in the promoter of *HTRA1* was reported to result in increased expression levels of HTRA1 mRNA and protein [22,23]. Our previous studies revealed that the SNP rs10490924 in exon 1 of the *ARMS2* gene is strongly associated with AMD and that rs11200638 had no significant impact on *HTRA1* promoter activity in various cell lines [24]. Another group reported an insertion/deletion polymorphism, NM_001099667.1: c.* 372_815del443ins54 in 3′-UTR of *ARMS2*, which is strongly associated with AMD; this variant deletes the polyadenylation signal and inserts a 54-bp AU-rich element, resulting in an unstable *ARMS2* transcript (Figure 1A) [25]. As strong linkage disequilibrium exists across the *ARMS2/HTRA1* region, genetic studies are likely insufficient to distinguish between the causal association of two genes with AMD. More recently, one report suggests that the haplotype carrying del443ins54 and rs11200638 affects *HTRA1* expression; this study used in vitro assays and RNA from human placenta samples [26]. Another recent report shows that del443ins54 does not lead to a decrease in the stability of *ARMS2* mRNA in lymphocytes [27]. We reasoned that human retina samples are necessary to clarify the impact of AMD-associated 10q26 SNPs on the biologic activity of *ARMS2* and *HTRA1* as reflected by their steady-state mRNA levels. Here, we report the results of our concurrent genotyping and qRT–PCR analyses of 35 retinas and 18 placenta samples.

## Methods

### Human tissues

Thirty-two retina tissues were collected from Caucasian individuals less than 6 h post-mortem and obtained from the National Disease Research Interchange (Philadelphia, PA), and three retina samples were from the Age-Related Histopathology Laboratory at the University of Alabama at Birmingham. Human placentas were obtained from the Labor and Delivery floor at the Hospital University of Pennsylvania within 4 h of delivery.

### Genotyping

Nucleic acids (total RNA with genomic DNA) were isolated from tissues using TRIzol (Invitrogen, Carlsbad, CA). A part of the sample was used to perform PCR amplifications with ExTaq (TaKaRa Bio USA, Madison, WI). The PCR amplified DNA was sequenced directly according to ABI BigDye terminator kit version 3.1 using ABI 3130xl Genetic Analyzer (Applied Biosystems, Foster City, CA). Primers for PCR amplification of 10q26 SNPs and sequencing are Fwd 5′-CCT TTG TCA CCA CAT TAT GTC C-3′ and Rev 5′-GCC TGA TCA TCT GCA TTT CTT AC-3′ for rs2736911 and rs10490924, Fwd 5′-ATC TGG ATT CCT CTC TGT CAC TG-3′ and Rev 5′-AAT GAA GTC CAA GCT TCT TAC CC-3′ for del443ins54, Fwd 5′-GAC GTG TGA AGG ATT CTA TTC GAA-3′ and Rev 5′-GCG TCC TTC AAA CTA ATG GAA CTT-3′ for rs11200638.

### qRT–PCR analysis and statistical methods

We checked RNA with gel and/or bioanalyzer. Reverse transcription (RT) was performed with Superscript II (Invitrogen) and oligo dT(20) primers per standard protocols [28]. Taqman gene expression assays were purchased for *HTRA1*, *GAPDH*, *HPRT1,* and 18S ribosomal *(r)RNA* and used on a 7900HT Fast Real-Time PCR System (Applied Biosystems) per the manufacturer’s instructions. SYBR Green PCR Master Mix (Applied Biosystems) was used for the *ARMS2* expression assay. The ARMS2 amplification primers were Fw 5′-GAT GGC AAG TCT GTC CTC CT-3′ and Rv 5′-TTG CTG CAG TGT GGA TGA TAG-3′. Real-Time qRT–PCR reactions were performed in quadruplicate. The relative quantification of the target transcript was performed using SDS2.0 and RQ manager software (Applied Biosystems). Gene expression levels were calculated using the 2^-ddCt^ method [29]. All experimental samples were normalized using human *rRNA* as an internal control. rRNA is a good internal control for gene expression in retinas, and showed uniform values. Other internal controls also showed similar results. The significance of the difference with a reference experiment was calculated using the Student's *t*-test, ANOVA (ANOVA) and linear regression.

## Results

We first examined whether there is any change in the expression of *ARMS2* and *HTRA1* between normal aged and AMD retinas without genotype constraints. The aged retinas were obtained from 20 unrelated individuals (average age=71.8 year), while the AMD group consisted of 12 retinas (average age=77.1 year). Gene expression was measured by real-time qRT–PCR and normalized to *rRNA* expression, with aged normal retinas as a reference. There was no significant difference in either *ARMS2* or *HTRA1* mRNA expression levels between the two groups (ARMS2, fold change average=1.051, p=0.487; HTRA1, fold change average=0.991, p=0.918). As predicted, the housekeeping genes (used as controls), *HPRT1* and *GAPDH*, did not show significant change in expression with age (fold change average=0.963, p=0.376 and fold change average=0.997, p=0.934), validating a high quality of RNA from the human retinas for qRT–PCR analysis.

To determine changes in RNA expression due to aging, we compared the gene expressions of normal young (average age=32.3 year, three retinas) and elderly (average age=75.5 year, six retinas) groups having common variants at all four genotyped loci (rs2736911, rs10490924, del443ins54, and rs11200638); no significant differences were obtained (ARMS2, fold change average=0.944, p=0.719; HTRA1, fold change average=1.078, p=0.634; HPRT1 fold change average=0.987, p=0.910 and GAPDH fold change average=0.974, p=0.778) (Figure 1B-E). Aged normal retinas were taken as a reference in this comparison. Additionally, we did not find any significant expression differences based on gender within the aged retinas.

To assess the impact of rs11200638 on *HTRA1* [23] and of del443ins54 on *ARMS2* mRNA [25], we determined the genotypes at the four 10q26 SNPs and measured the expression levels of *ARMS2* and *HTRA1* in all 35 retinas using real-time qRT–PCR. First, we conducted general tests for all samples to study the association between gene expression and the four polymorphisms regardless of disease and age status. ANOVA and linear regression were performed respectively on free and additive models for genotypes. There is no significant gene expression difference (p>0.05). A similar result was obtained for analysis in case or control group only. Furthermore, we analyzed the expression data by stratifying on different genotypes. Heterozygous rs10490924, del443ins54, and rs11200638 did not affect the mRNA levels of *ARMS2* and *HTRA1* genes (*ARMS2*, fold change average=0.989, p=0.944; *HTRA1*, fold change average=0.985, p=0.916). No expression changes were detected even in individuals homozygous for these SNPs (*ARMS2*, fold change average=0.926, p=0.589; *HTRA1*, fold change average=1.197, p=0.302; Figure 1B,C). Additionally, retinas heterozygous or homozygous for the rs2736911 SNP did not show a significant change in expression (heterozygote, ARMS2, fold change average=1.046, p=0.785; HTRA1, fold change average=1.184, p=0.435) (homozygote, ARMS2, fold change=0.706; HTRA1, fold change=1.287; Figure 1B,C). Although our results for homozygous rs2736911 are represented by only one retina, it does not appear to affect the expression; this SNP causes nonsynonymous R38X alteration in ARMS2 and it is not reportedly associated with AMD risk. No significant differences in expression were observed for *HPRT1* and *GAPDH* (p>0.05; Figure 1D,E).

**Figure 2:**
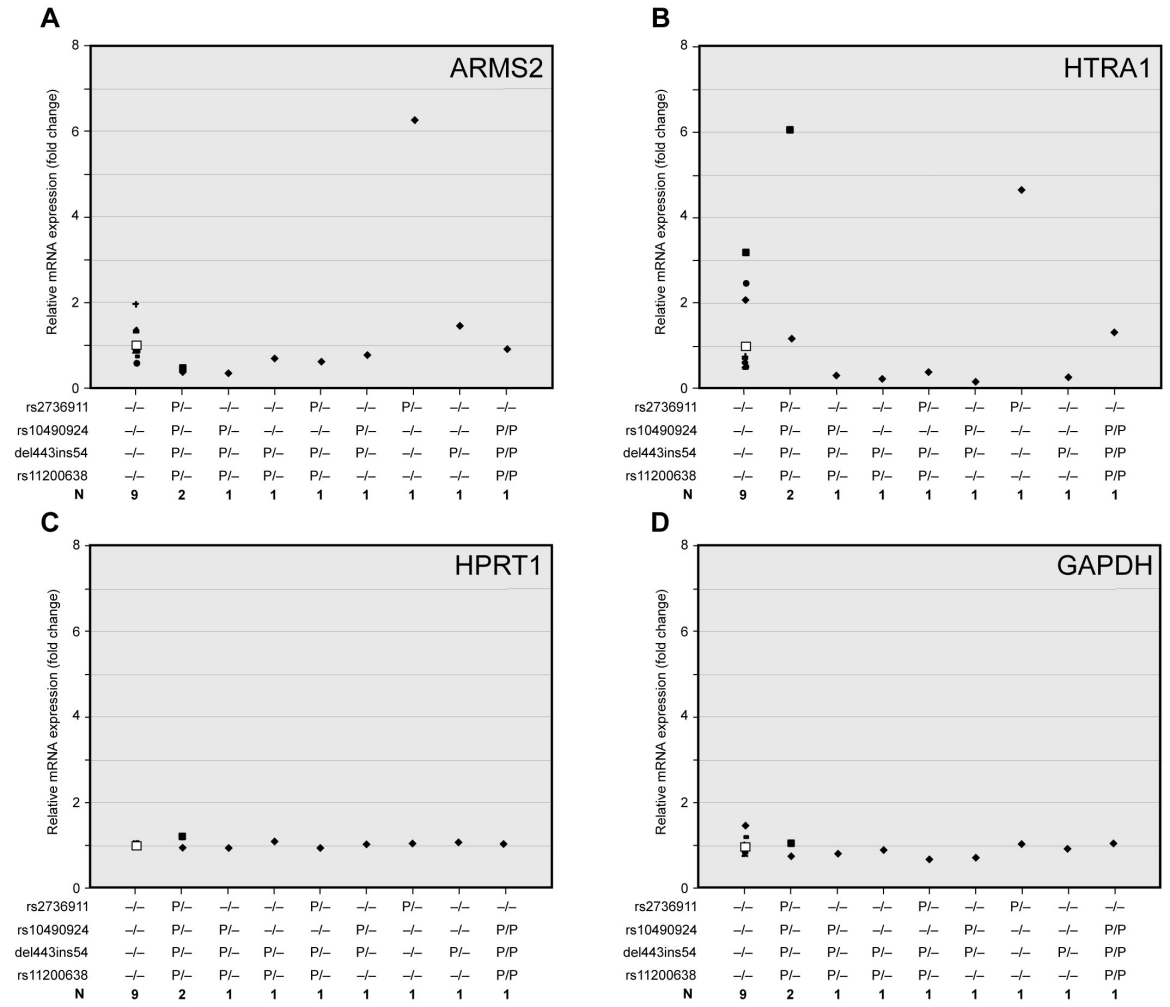
Relationship of *ARMS2* and *HTRA1* expression in the human placenta to the genotypes at 10q26. We show the effect of AMD-associated variants near *ARMS2* and *HTRA1* genes on *ARMS2* and *HTRA1* expression in human placenta samples. **A**-**D**: The fold change in expression of the target genes (*ARMS2, HTRA1, HPRT1*, and *GAPDH*) relative to the internal control gene (*rRNA*) were correlated to genotypes of corresponding placenta samples. Real-time qRT–PCR reactions were performed in quadruplicate with Taqman gene expression or SYBR Green assays. The fold change average in the expression of the target gene was calculated using the 2^-ddCt^ method; ddCt=(C_t_,Target - C_t_,rRNA)_experiment_ - (C_t_,Target - C_t_,rRNA)_reference_. Gene expressions in wild-type (having common variants at all four genotyped loci) placentas were used as a reference. cBlack unique symbols (e.g., triangle, square, diamond, dash, X’s cross, circle) indicate individual sample changes, and white square shows the average. Abbreviations: N represents number of samples with given genotypes; P represents polymorphism (P/P represents homozygous; P/- represents heterozygous change. Variants are shown in the left column).

We then compared expression between six aged normal and four AMD (age average=81.5 year) retinas that did not carry the polymorphic variants at the four 10q26 loci tested. *ARMS2* and *HTRA1* expression did not reveal a significant difference (*ARMS2*, fold change average=0.970, p=0.793; *HTRA1*, fold change average=1.104, p=0.585; *HPRT1*, fold change average=0.924, p=0.296; *GAPDH*, fold change average=0.929, p=0.307; Figure 1B-E). Further analysis showed no significant differences in *HTRA1* and *ARMS2* expressions in AMD retinas that were heterozygous for rs10490924, del443ins54, and rs11200638 (*ARMS2*, fold change average=0.943, p=0.771; *HTRA1*, fold change average=0.953, p=0.769), and heterozygous rs2736911 (*ARMS2*, fold change average=1.133, p=0.383; *HTRA1*, fold change average=1.084, p=0.685) compared to normal retinas with the common alleles (Figure 1B,C). While there was only one AMD retina homozygous for del443ins54 or rs11200638, the expression levels exhibited no appreciable change from normal retinas (*ARMS2*, fold change=1.001; *HTRA1*, fold change=1.084). *HPRT1* and *GAPDH* expression in these retinas did not reveal a significant difference (p>0.05; Figure 1D,E). We also evaluated the expression of *ARMS2* and *HTRA1* in human placentas. However, the expression levels of these two genes demonstrated wide variations that could not be explained by their genotype (Figure 2A,B). Interestingly, the two housekeeping genes (*HPRT1* and *GAPDH*) did not manifest the large variations in expressions observed for *ARMS2* or *HTRA1* (Figure 2C,D).

## Discussion

Gene expression changes have been investigated in multiple species and scenarios to study the genetic basis of variations in gene regulation [30-32]. The binding of transcriptional factors to a specific sequence constitutes a key aspect of gene regulation. Other means of regulating gene expression include DNA methylation, alternative splicing, and mRNA stability [33]. Genetic variations (DNA polymorphisms) are known to be associated with phenotypic variation and may alter gene expression patterns [34].

A genetic locus at chromosome 10q26 provides strong susceptibility to AMD; specifically, two AMD-associated polymorphisms near *ARMS2* and *HTRA1* genes have been suggested to alter the gene expression of either one or the other gene [23,25,26]. The investigations of associated polymorphisms and their impact on expression levels have been elusive and contradictory. To examine this issue further and in the context of aging and AMD retinas, we concurrently obtained genotypes of human normal and AMD retinas and examined whether specific haplotypes or polymorphisms at 10q26 might be correlated to steady-state transcript levels of *ARMS2* or *HTRA1*. In our study, none of the four polymorphisms (rs2736911, rs10490924, del443ins54, and rs11200638) either as homozygotes or heterozygotes or their associated haplotypes significantly affected the *ARMS2* or *HTRA1* gene expression levels. One study recently reported that haplotypes with del443ins54 and rs11200638 variants have an effect on *HTRA1* and *ARMS2* expression in genotyped human placentas [26]. However, another report presented that the del443ins54 variant does not change *ARMS2* mRNA stability in lymphocytes [27], which is consistent with our data from retina samples. In our study, *ARMS2* and *HTRA1* gene expression levels in the placentas exhibited wider variations than in retinas, thus making it difficult to draw a definitive conclusion. While all our retinas were collected from Caucasian subjects, no ethnic information was available for our placenta samples. The wide variations in *ARMS2* and *HTRA1* may be a reflection of ethnic differences; curiously however, the housekeeping genes (*HPRT1* and *GAPDH*) did not exhibit such differences in expression. The two previous studies examined the effects of del443ins54 and rs11200638 variants in transfected human embryonic kidney cells or transfected mouse RPE [25,26]; however, there is a poor homology in the human and mouse DNA sequence about 13kb upstream from the *HTRA1* transcriptional start site, and the *ARMS2* gene, including del443ins54, is primate specific. We suggest that *ARMS2* and *HTRA1* mRNA expression and stability are regulated via distinct mechanisms in the retina compared to other tissues. It should be noted that the del443ins54 variant sequences in our samples are similar to those reported previously [25,35], and no additional changes are identified as reported recently [27].

Interestingly, *ARMS2* and *HTRA1* expression levels in a single human retina sample that is homozygous for the ARMS2-R38X variant (rs2736911) are similar to those of other genotypes. This variant is predicted to produce a truncated ARMS2 protein. We must note that AMD is a multifactorial disease with numerous susceptibility loci [18]; therefore, the altered ARMS2 expression or function alone will not be sufficient to cause AMD. The *ARMS2* gene exists only in primates but not in other vertebrates, thus it is unlikely to have an essential or critical role in retinal physiology. We hypothesize that ARMS2 has a unique supportive function in photoreceptors and/or retinal pigment epithelium of primates.

In conclusion, our studies do not support the direct association between the genotype of rs11200638 and *HTRA1* mRNA levels, and between the del443ins54 variant and *ARMS2* mRNA levels in human retina samples we have analyzed. We suggest that comprehensive investigations at the protein and function levels, not only the protein expression, will be necessary to further clarify the roles of ARMS2 and/or HTRA1 in AMD etiology. Such studies will require well characterized and specific antibodies for ARMS2 and HTRA1 and investigations with human (or primate) tissues/samples carrying distinct AMD-associated genotypes.
